# Parenteral–Oral Immunization with Plant-Derived HBcAg as a Potential Therapeutic Vaccine against Chronic Hepatitis B

**DOI:** 10.3390/vaccines7040211

**Published:** 2019-12-09

**Authors:** Marcin Pyrski, Adam Aron Mieloch, Adam Plewiński, Aneta Basińska-Barczak, Aleksandra Gryciuk, Piotr Bociąg, Marek Murias, Jakub Dalibor Rybka, Tomasz Pniewski

**Affiliations:** 1 Institute of Plant Genetics, Polish Academy of Sciences, Strzeszyńska 34, 60-479 Poznań, Poland; mpyr@igr.poznan.pl (M.P.); abas@igr.poznan.pl (A.B.-B.); a.gryciuk@tlen.pl (A.G.); bociagpiotr@tlen.pl (P.B.); 2 Center for Advanced Technology, Adam Mickiewicz University, Uniwersytetu Poznańskiego 10, 61-614 Poznań, Poland; adamie1@amu.edu.pl (A.A.M.); adam.plewinski@amu.edu.pl (A.P.); 3 Faculty of Chemistry, Adam Mickiewicz University, Uniwersytetu Poznańskiego 8, 61-614 Poznań, Poland; 4 Department of Toxicology, Poznan University of Medical Sciences, Dojazd 30, 60-631 Poznań, Poland; marek.murias@ump.edu.pl

**Keywords:** chronic hepatitis B, HBcAg, immune response, IgG isotypes, parenteral–oral immunization, plant-derived antigen, therapeutic vaccine

## Abstract

Chronic hepatitis B (CHB) is the cause of severe liver damage, cirrhosis, and hepatocellular carcinoma for over 240 million people worldwide. Nowadays, several types of treatment are being investigated, including immunotherapy using hepatitis B core antigen (HBcAg) assembled into highly immunogenic capsid-like particles (CLPs). Immunogenicity of plant-produced and purified HBcAg, administered parenterally or intranasally, was previously reported. In this study, a novel parenteral–oral vaccination scheme is proposed using plant-derived HBcAg preparations. The antigen for injection was obtained via transient expression in *Nicotiana benthamiana*. HBcAg-producing transgenic lettuce was lyophilized and used as an orally delivered booster. The intracellular location of plant-produced HBcAg CLPs implies additional protection in the digestive tract during oral immunization. BALB/c mice were intramuscularly primed with 10 µg of the purified antigen and orally boosted twice with 5 or 200 ng of HBcAg. A long-lasting and significant systemic response after boosting with 200 ng HBcAg was induced, with anti-HBc titer of 25,000. Concomitantly, an insignificant mucosal response was observed, with an S-IgA titer of only 500. The profile of IgG isotypes indicates a predominant Th1 type of immune response, supplemented by Th2, after injection–oral vaccination. The results demonstrate that a low dose of parenteral–oral immunization with plant-derived HBcAg can elicit a specific and efficient response. This study presents a potential new pathway of CHB treatment.

## 1. Introduction

Hepatitis B virus (HBV) still remains the major cause of liver diseases, especially in developing countries [1]. Despite widely implemented national obligatory prevention programs using effective vaccines based on HBV surface antigen (HBsAg), eradication of HBV is still a vision. Because of problems with the management of HBV infection, emergence of HBV mutants and considerable extent of non-responsiveness to vaccines, an estimated over 2 billion people suffer from hepatitis B. From these, 10%–15%, especially infants, remain chronically infected, which is one of the causes of the maintained reservoir of the virus [1,2]. Chronic hepatitis B (CHB) is a type of HBV infection, where the presence of the virus persists for more than six months and may extend to produce a life-long disease. Persistent infection may disrupt physiological functions of the liver. Sustained malfunctioning may lead to progressing organ damage, which in turn can result in liver fibrosis and, subsequently, cirrhosis and hepatocellular carcinoma (HCC). It is estimated that at least 240 million people are living with CHB, and more than 600 thousand patients die annually from HCC and other CHB complications [3,4].

Nowadays, CHB treatments are based on nucleoside analogues or IFN-α; however, their cure rates remain low [2,3,4]. Therefore, new therapeutic routes are extensively investigated and are also in clinical trials [2,5]. Current immunotherapies are based on restoration of the anti-HBV cellular response using immunomodulators or direct antiviral agents including therapeutic vaccines [6,7]. Hepatitis B core antigen (HBcAg) assembled into highly immunogenic capsid-like particles (CLPs) is proposed as the main component of a potential therapeutic vaccine against CHB [5,8]. HBcAg CLPs activate both cellular and humoral immune responses after parenteral or mucosal delivery [8,9].

HBcAg is produced in well-established standard expression systems such as bacteria, yeasts, and mammalian cells. Then, it is delivered mostly via parenteral or intranasal routes; thus, it has to be purified and delivered using supplementary equipment. This generates additional costs, not to mention safety issues. Alternatively, plants offer several benefits as producers and carriers of vaccines [10]. HBcAg can be obtained at low cost and in high yields using plant expression systems [9,10,11]. In our previous work, HBcAg produced in transgenic tobacco elicited a specific, anti-HBc response in mice. Obtained results were comparable to a commercially available, yeast-derived antigen and induced mainly the Th1 response, together with some Th2 polarization [12]. In turn, the oral route represents one of the most cost-effective and safe ways to administer therapeutics and vaccines, including HBV antigens [13]. Following the above-mentioned premises, HBcAg production in transgenic plants for potential use as an oral vaccine seems a reasonable and valid approach. However, the main challenge for oral immunization remains the adjustment of an efficacious vaccine formulation and administration regime to induce active immunity, instead of oral tolerance [14]. Recently, we have developed a low-dose oral immunization protocol for a potential HBsAg-based plant-derived prophylaxis booster vaccine against hepatitis B [15]. Yet, efficacious combat against HBV also demands a synergistically applied therapeutic vaccine for CHB treatment.

Hence, the purpose of this study was to prepare and evaluate the immunogenicity of plant-produced HBcAg as a parenteral–oral vaccine. Firstly, purified HBcAg obtained via transient expression in *Nicotiana benthamiana* plants was injected as the priming dose. Subsequently, lyophilized transgenic lettuce containing HBcAg was administered intragastrically as the booster in two tested doses. Mucosal and systemic immune responses triggered by injection–oral immunization were determined. Additionally, the type of systemic response was defined.

## 2. Materials and Methods

### 2.1. Transient Expression in Nicotiana benthamiana

Plants for agroinfiltration were cultivated in a walk-in growth chamber at 16 °C under 5–6 klx light intensity and a 16/8 photoperiod. The procedure of transient expression was performed as described previously with the use of the pEAQ-HBc vector [16]. In the experiments, two *Agrobacterium tumefaciens* strains, LBA4404 or EHA105, were used. After 10 d following agroinfiltration, HBcAg was extracted and purified as described previously with minor modifications, which included a 60% sucrose cushion and centrifugation at 30,000× *g* at 4 °C for 30 min [12].

### 2.2. Lettuce Stable Transformation

The pKHBCBAR vector carrying the coding region of HBcAg subtype *ayw4* (GenBank accession No. Z35716) was constructed as described previously [12] and used for obtaining transgenic lettuce via *Agrobacterium*-mediated transformation as reported earlier [17].

### 2.3. Microscopic Observations of Plant-Expressed HBcAg

Leaf pieces of approximately 1 cm^2^ were fixed for 24 h at 4 °C in a mixture of 4% paraformaldehyde (Polysciences, Hirschberg an der Bergstrasse, Germany), and 0.5% glutaraldehyde (both Polysciences Europe GmbH, Hirschberg an der Bergstrasse, Germany) in PBS buffer, followed by dehydration in standard series dilutions of ethanol and embedded in Technovit 8100 resin (Kulzer GmbH, Wehrheim, Germany) according to the manufacturer’s protocol. Samples were cut on a rotary microtome (Leica Microsystems GmbH, Wetzlar, Germany) to slices 5 µm thin and mounted on microscope slides coated with poly-l-lysine (Merck KGaA, Darmstadt, Germany, formerly Sigma, St. Louis, MO, USA). Subsequently, slides were blocked in 2% BSA in PBS for 3 h and then incubated overnight at room temperature with primary anti-HBc polyclonal rabbit antibody (Cat. No. LS-C67451, LSBio, Seattle, WA, USA). After washing three times with PBS buffer, the samples were incubated with goat anti-mouse secondary antibody (H+L) Cy3-conjugated (AB_2534030, Thermo Fisher Scientific, Waltham, MA, USA) for 1.5 h at room temperature. After washing with PBS buffer and deionized water, slices were coated with Citifluor™ AF1 (Electron Microscopy Science, Hatfield, PA, USA), closed with the cover glass, and sealed using nail polish. Images were obtained using a confocal scanning laser microscope FV1200 (Olympus, Tokyo, Japan).

### 2.4. Lyophilization

Leaves from the lettuce plants with the highest HBcAg content were frozen in liquid nitrogen, crushed, and placed in a chamber at −35 °C. Lyophilization was performed in a BETA 1-16 freeze-dryer (Martin Christ Gefriertrocknungsanlagen GmbH, Osterode am Harz, Germany) at 0.2 mbar at 20 °C for 20 h (primary drying) followed by 22 °C for 2 h (secondary drying). Lyophilized tissue was powdered in a coffee mill chilled on ice and stored in the dark at 4 °C with silica gel as a desiccator. Residual moisture in the tissue was determined using the gravimetric method [18]. The concentration of HBcAg was assayed directly after freeze-drying (day 1) and after 3, 6, 9, and 12 months of storage.

### 2.5. Mouse Immunization

The mouse immunization trial was conducted in accordance with the standards of the Council Directive 86/609/EEC and approved by the Local Bioethical Commission in Poznań, Poland (Decision No. 77/2017). Female BALB/c mice (AnimaLAB, Poznań, Poland) 10 weeks old with bodyweights of approximately 20 g were used in this study. Two weeks after the acclimatization period, mice were divided into the following groups: 2 experimental groups of 10 mice each, 2 reference groups of 5 mice each, a negative control of 5 mice, and a naïve group of 5 mice. Priming was conducted in the experimental and reference groups by intramuscular injections (femoral muscle) of 10 µg plant-derived HBcAg, delivered in a total volume of 200 µL sterile PBS, using an insulin needle (0.33 × 12 mm). At four and ten weeks after priming (days 29 and 70), the experimental groups were intragastrically administered, using a feeding needle (Kent Scientific Corporation, Torrington, USA), the plant lyophilisate containing HBcAg at 200 and 5 ng, ‘high’ and ‘low’ doses, respectively, suspended in 100 µL of PBS. Reference mice were administered the control lyophilisate as above in the amounts equal to the experimental doses. Blood samples and feces were collected prior to the first immunization (pre-immune, day 3) and on days 28, 69, 86, and 100. Blood samples from the facial vein were centrifuged at 3000 rpm at 4 °C for 15 min, and sera were collected. S-IgA were extracted from the feces as described previously [17].

### 2.6. ELISA Tests

The contents of CLP-assembled HBcAg in raw plant tissue, in the lyophilisate, and after purification were determined using a previously established sandwich ELISA [12]. Titers of anti-HBc antibodies, including S-IgA in feces, total antibodies, and IgG isotypes in serum, were determined as previously described [12].

### 2.7. Statistical Analysis

**Figure 1:**
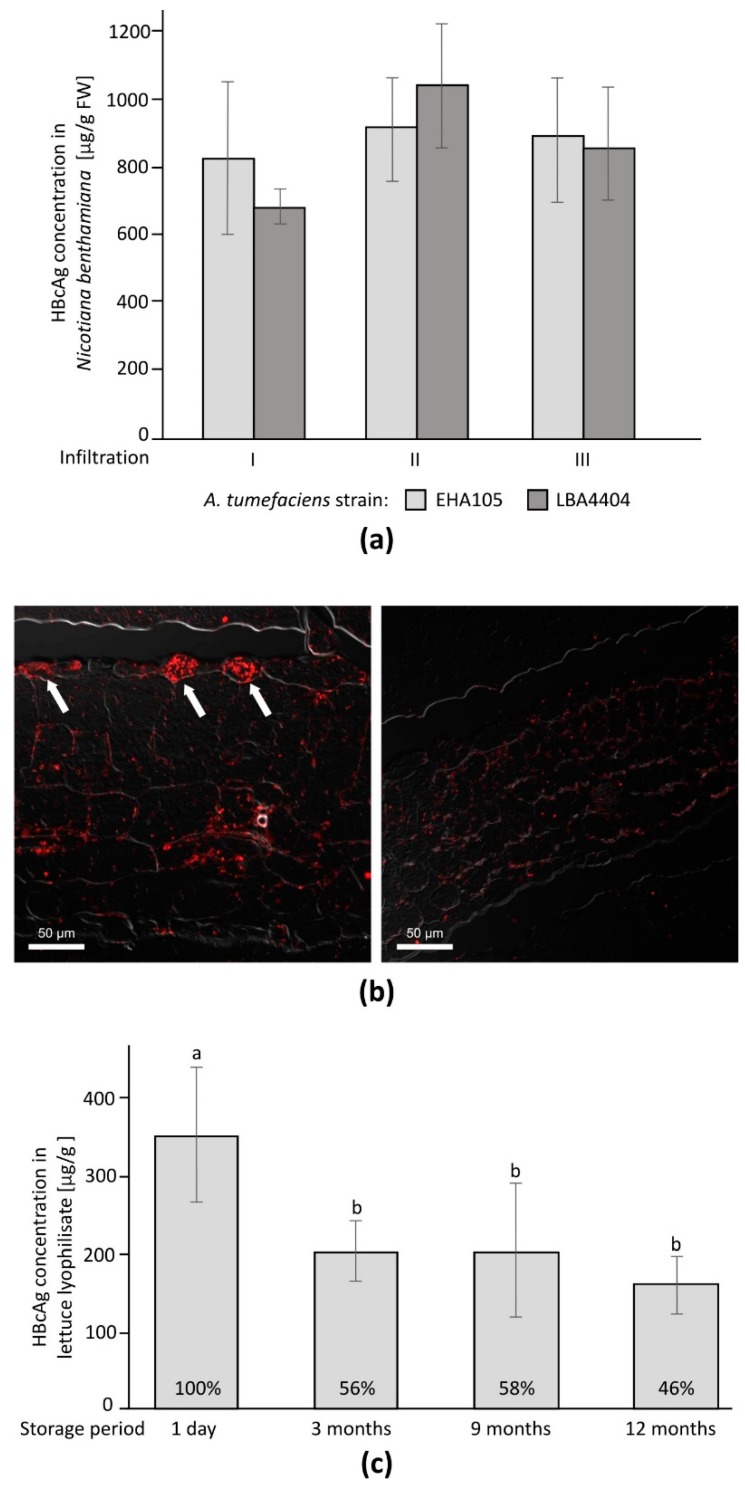
Production of plant-derived HBcAg: (**a**) yield of transiently expressed HBcAg; (**b**) plant tissue expressing HBcAg (left) in comparison to the control (right), arrows indicate sites of particularly large antigen deposition; (**c**) HBcAg preservation in lyophilized tissue in absolute (µg/g) and relative (percent) units, significant differences marked by small caps letter indexes.

**Figure 2:**
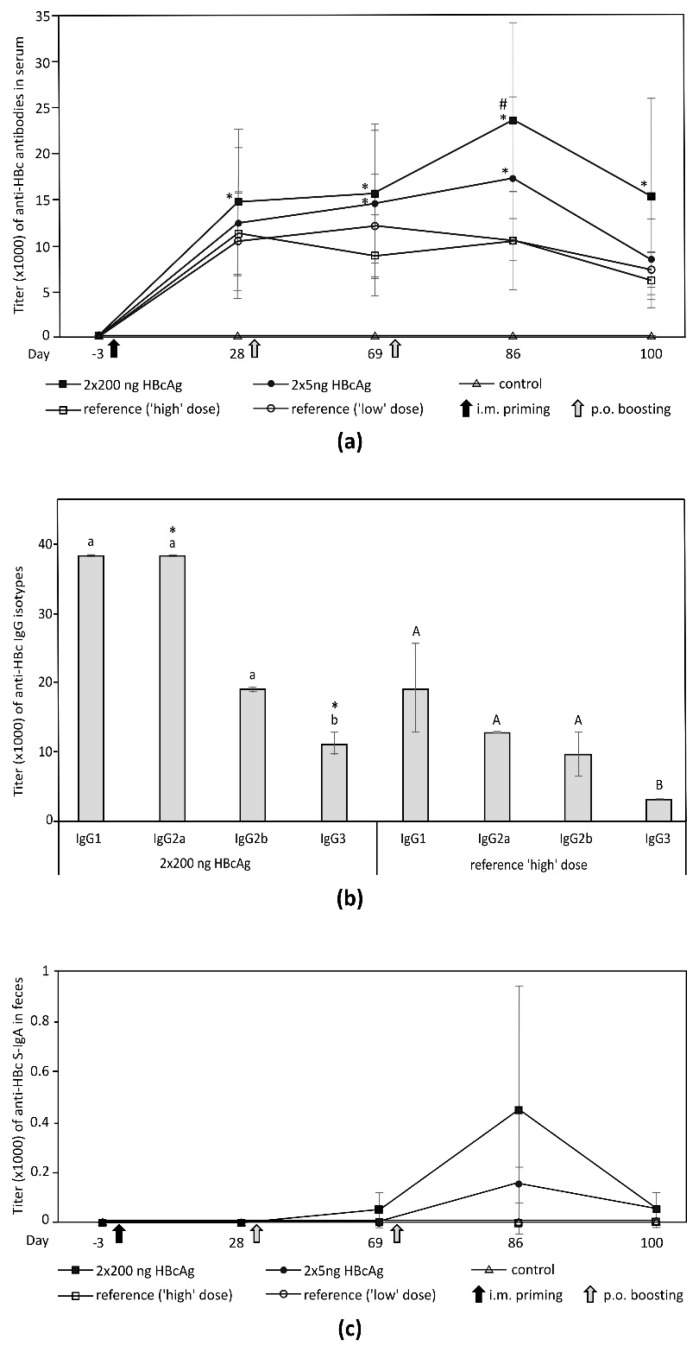
Immunogenicity of plant-derived HBcAg administered via intramuscular priming using purified antigen and oral boosting using antigen in lyophilized tissue. Mice in reference groups were given control lyophilisate, while these in the control group were delivered PBS and control lyophilisate. (**a**) Systemic humoral response, significant anti-HBc titer vs. pre-immune and priming marked by asterisks and hashes, respectively; (**b**) Anti-HBc IgG profile 16 d after the 2nd oral boosting (day 86) for the group orally boosted 2 × 200 ng HBcAg (significant response) and its reference. Antibody titers expressed as means from three assays of pooled sera (10 or 5 mice per experimental or reference group, respectively). Titers represent the highest dilution of serum required to yield the cut-off, calculated as the mean for pre-immune sera plus tripled SD. Intragroup significant differences indicated by small caps and capitals indexes for the experimental and reference group, respectively. Significant differences for a given IgG isotype between the experimental and reference groups are marked by asterisks; (**c**) mucosal response-differences were insignificant. Note: results for naïve mice were actually the same as for control; thus, they are not shown for clarity of presentation.

Changes in the HBcAg content in stored lyophilisate were analyzed using ANOVA for repeated measures (Figure 1c). Inter- and intragroup analyses of the immune responses at consecutive time points (Figure 2a,c), as well as the intragroup comparison of IgG isotypes for day 86 (maximum response, Figure 2b), were performed using a Kruskal–Wallis ANOVA with a test for multiple comparisons of mean ranks. Titers of particular IgG isotypes in the experimental and corresponding reference HBcAg groups were compared using the Mann–Whitney U test (Figure 2b). Differences were considered significant at *p* ≤ 0.05. Statistical analysis was performed using the Statistica 8.0 statistical software package (StatSoft Inc., Tulsa, OK, USA).

## 3. Results

### 3.1. Transient Expression of HBcAg in Nicotiana benthamiana

After 7–8 d post infiltration, accumulation of HBcAg in the plant tissue reached the highest plateau level. Vacuum-assisted infiltration resulted in a lower HBcAg content, approximately 0.2 mg/g of fresh weight (FW) in comparison to 0.6–1 mg/g FW for syringe-based infiltration. There were no significant differences in HBcAg accumulation after infiltration with the two *Agrobacterium* strains (Figure 1a). Because of a simpler culturing procedure and less severe symptoms on the infiltrated leaves, strain LBA4404 was used for further experiments. After purification via sucrose cushion centrifugation, the concentration of HBcAg reached 400 µg/mL.

### 3.2. HBcAg Expression in Transgenic Lettuce

There were 23 transgenic lettuce plants (T0 generation) after *Agrobacterium*-mediated transformation with the pKHBCBAR vector, as verified by PCR (not shown). Most plants expressed HBcAg at a level of several tens of µg/g FW. Production of HBcAg in plants of the T1 generation dropped to over a dozen of µg/g FW. However, these amounts were sufficient for the studies on lyophilization and immunogenicity of a plant-derived HBcAg. Transformants with the highest and also the most stable HBcAg expression sustained during the lifecycle were selected for further research.

### 3.3. Observation of HBcAg CLPs in Plant Tissue

HBcAg in the plant tissue was detected via immunochemical localization. Multiple specific signals were observed in the leaves of plants expressing HBcAg in comparison to control plants (Figure 1b). Larger depositions of HBcAg CLPs were localized mainly in the upper epidermis. The signal clusters were localized within the whole cytoplasm, not in particular cell compartments.

### 3.4. Lyophilization and Material Stability

The HBcAg concentration in the plant tissue used for lyophilization was 18.3 µg/g FW and during the process slightly decreased to 97.8% considering water loss. The absolute content in dry material directly after lyophilization was 344.6 µg/g. The analysis of HBcAg stability in the lyophilized material stored at 4 °C after 3 months showed a decrease in the HBcAg content by almost 50%, but then it remained stable for up to 12 months after lyophilization (Figure 1c). Moisture of the lyophilisate was maintained below 3.5% throughout the whole storage period.

### 3.5. Immunogenicity of Plant-Derived HBcAg

No alterations in condition or behavior were observed among the experimental, reference, control, or naïve mouse groups. Also, bodyweight changes (increase by 6%–10%) differed insignificantly among groups. Specific immune responses after the priming injection were induced only in the experimental and the reference groups (Figure 2a). After the first and second oral immunization with the lyophilisate, the titer of anti-HBc antibodies was significantly increased in comparison to that at pre-immune levels. A significant boosting effect was observed only in the group administered the ‘high’ dose (2 × 200 ng) of the plant-derived HBcAg (Figure 2a). The profile and titers of anti-HBcAg IgG isotypes corresponded to the general humoral response. Mostly IgG1 and Ig2a were elicited, yet induction of IgG2b and IgG3 was also noticeable. In the ‘high’-dose group the titer for each IgG isotype was higher, but significant differences were observed only for IgG2a and IgG3 in comparison to its reference group. The titer of IgG3 was significantly lower than for any other isotype (Figure 2b). For the ‘low’-dose group (HBcAg 2 × 5 ng) there were no significant differences between the experimental and respective reference groups (data not shown). A mucosal immune response was observed only in the experimental groups. However, mean titers of S-IgA in feces were ≤500 and insignificant versus pre-immune levels (Figure 2c).

## 4. Discussion

The validity of exploitation of plants as a system for the production of parenteral and oral vaccines and therapeutic proteins has been demonstrated in many reports [13,19,20]. In this study, we have proven that plant-derived HBcAg can be effective as a parenteral primer and oral booster vaccine.

HBcAg used for priming was transiently expressed in *Nicotiana benthamiana* at up to 1 mg/g FW, similarly to the protocols reported elsewhere [9,11,21], and purified using a simple method of sucrose cushion centrifugation. The antigen concentrations in the transgenic lettuce were much lower, which is typical of stable transformation; however, the amount of suitable plant material could be easily multiplied by clonal propagation [22]. Lyophilization provided a 19-fold higher concentration of the antigen produced in transgenic lettuce and the reduction of tissue volume for oral immunization. The HBcAg content in the lyophilized tissue initially dropped, but after the third month of storage it remained stable and on a considerable level of approximately 50%, for at least one year of cold storage, which may be considered as a good starting point for optimization research. Analogously to the HBV surface antigen, HBcAg stability most probably may be further increased by the addition of appropriate protectants and adjustment of storage conditions to facilitate its storage at elevated temperatures, which would be advantageous in comparison to liquid formulations [23]. Microscopic immunochemistry showed a high intracellular accumulation of the HBcAg CLPs. Such bioencapsulation may subsequently provide additional protection from degradation in the digestive tract, even though HBcAg is relatively stable in the acidic environment of the stomach [24]. Hence, considering both findings, orally administered plant-associated HBcAg passes through the intestine, activating Gut Associated Lymphoid Tissue (GALT), as also described for other antigens [25,26].

Immunogenicity of the HBcAg obtained via bacterial recombination (e.g., *Salmonella* sp. or by transient expression in plants) when delivered through mucosal membranes was confirmed previously [9,27,28,29]. This study demonstrates for the first time that an orally administered lyophilized plant tissue bearing low concentrations of the HBcAg, and without any addition of exogenous adjuvants, was sufficient for an effective immunization. Vaccination with HBcAg through the mucosal membrane of the intestine can be considered as progress since it has been known that orally delivered antigens, in general, poorly induce immunity, favoring mostly oral tolerance. Antigens assembled into oligomers or Virus-Like Particles (including CLPs) are more immunogenic than soluble ones, yet conditions of their application still affect the GALT response, which can develop into local or systemic immunity or oral tolerance [30,31]. The latter can be acquired either via clonal deletion or anergy, when an antigen is at a high dose, or by regulatory T cell induction for a low antigen dosage [30]. Nevertheless, a very low antigen dosage or long intervals between administrations can induce an active immune response [31,32]. Based on this finding, as well as our previous experiments, we have tested here as low as nanogram doses, 5 or 200 ng of plant-associated HBcAg, administered as a booster vaccine in relatively long intervals (i.e., four and ten weeks) after intramuscular priming. From the two doses tested, the higher induced a significant response, not only versus pre-immune but also in comparison to only primed mice, clearly indicating the boosting effect. The respective mechanism is assumed to be the same as that proposed for the low-dosed HBV surface antigen [15]. According to that hypothesis, parenteral priming and oral boosting act synergistically. Circulating Th cells, previously sensitized by parenterally delivered antigen, may be subsequently stimulated in mesenteric lymph nodes by an antigen from the intestine mediated by M cells and Antigen Presenting Cells (APCs). In this way, a systemic response might be increased with concomitant suppression of oral tolerance, further reduced by the low antigen dosage and long intervals between immunizations [15]. However, in comparison to that study on injection–oral immunisation with HBsAg, the period before the first oral boosting was slightly shortened in our study (i.e., four instead of six weeks). This interval was a type of compromise between vaccination protocols from preceding experiments. While oral boosting using plant lyophilisate bearing the HBV surface antigen was effective when performed as long as six weeks post priming, parenteral immunization using plant-derived HBcAg suggested that two weeks were sufficient to achieve sufficient titers of anti-HBc prior boosting. In the presented study, the titer of anti-HBc four weeks after priming was similar to that observed previously [12], but it was significant only for the 2 × 200 ng p.o. immunized group. In our opinion, this should be considered in the context of naturally occurring variations in mice regarding immunological responsiveness. More essential was that differences after priming between particular mouse groups were insignificant, and the significant differences among both experimental versus reference groups were revealed only after oral boosting.

Finally, in the result of adopted immunization protocols via injection and two low-dose oral boostings, the titer of anti-HBc in serum reached the value of 25,000, which is considerably higher than those observed in previous trials using a high-dosed antigen (i.e., 100–500 µg) [9]. Moreover, the profile of IgG indicates that the HBcAg of plant origin elicited predominantly the Th1 type of immune response but with a noticeable Th2 response after the combined injection–oral administration. The titers of IgG2a and IgG1 were comparable. The titers of IgG2b and IgG3 were also relatively high, as their respective values reached approximately one-half or one-third of that for IgG1. The titers of IgG2a and IgG3 were significantly higher in the experimental group (2 × 200 ng) than in the respective reference group. In mice, a potent production of IgG2a antibodies indicates a strong antiviral response [33]. The titer of total anti-HBc antibodies finally decreased (day 100), which was observed also when HBcAg was delivered exclusively via injection [12]. Yet, the induced Th1 response implied development of a cellular response, which is considered as the main mechanism of CHB cure [6,34]. The observed course of immune response may also suggest that, for HBcAg, as an example of an antigen inducing mostly the Th1 response, two or more low-dosed oral boostings elicit significant immunity, while for Th2-inducing antigens, for instance HBsAg, a single oral boosting might be sufficient [14,34]. Both above hypotheses could be verified in other studies though.

In comparison to the anti-HBc in serum, production of S-IgA was very low, as the respective maximum titer was only 500. These results indicate a strong systemic response triggered by the low-dose injection–oral immunization, while the mucosal response, undesirable from a therapeutic perspective, is practically negligible. Although the active response of the GALT is predominantly mucosal immunity, there are data indicating induction of a systemic response following appropriate antigen dosage [31]. Hence, we presume that the trace induction of anti-HBc S-IgA here may have resulted from an adopted immunization protocol comprising the prime injection and low-dosed oral boosting performed in long intervals, which is analogous to immunization with HBsAg [15]. This supposition can be supported by other reports, which showed that exclusive oral immunization or higher or frequent dosage of antigens evoked polarization of the GALT response toward significant mucosal immunity, sometimes over a systemic one [17,35,36]. Moreover, in this context, the induction of a significant systemic response by orally administered plant-derived HBcAg without any exogenous adjuvant can be considered an additional benefit. On the other hand, a possible increased uptake of HBcAg by intestinal mucosal APCs or other positive effects exerted by an adjuvant cannot be excluded; thus, a range of these agents could be screened [37,38].

## 5. Conclusions

We previously demonstrated that successful parenteral immunization with plant-derived HBcAg could be elicited by a relatively low dosage at 10 µg [12]. In this study, we proved that an oral booster immunization with plant-associated low-dosed HBcAg was also feasible and similar to the HBV surface antigen [15]. What is more, plant-produced HBcAg administered via an injection–oral immunization regime elicited predominantly a Th1 response, supported by Th2, which is particularly desired for CHB therapy [8]. The presented research was preliminary, yet the above-mentioned findings constitute a solid basis for further optimization studies on the plant-derived therapeutic vaccine against CHB. Immunogenicity of a plant-derived HBcAg-based vaccine can be enhanced, both by improved preparation and formulation of the components and by adjusted details of immunization protocol, especially dosage and schedule. Also, the immune response has to be meticulously analyzed, followed by evaluation of possible CHB therapeutic effects in transgenic mice. Assuming efficacy of the injection–oral vaccination, a plant-derived CHB therapeutic vaccine could be subsequently tested in clinical trials and then used for human immunization, although similarly to other tested CHB vaccines, probably not until the next decade [2]. Nevertheless, a potential plant-derived CHB vaccine would substantially improve public health, especially in developing countries. The main rationale for the use of plants as the source of both injection and oral vaccines is connected with its low-cost, scalable, and safe production [13,19,20,39]. Thanks to effective oral boosting, additional costs associated with HBcAg purification and delivery via injection could be reduced to an indispensable minimum (i.e., priming only). Furthermore, in the case of oral components, the cost of vaccine manufacturing may be reduced due to the minimal processing requirement of lyophilization. In this way, sustainability of immunization and independence from a foreign supply may become the foundation of national programs of a CHB cure using plant-derived therapeutic vaccines. This could contribute to decrease the HBV reservoir and subsequently to facilitate prevention of hepatitis B and associated diseases. The presented study, together with the others, indicates that plant-expressed proteins could be successfully used in medicine as a cost-effective alternative for current therapeutics [13,40].
